# The genetics of gout: towards personalised medicine?

**DOI:** 10.1186/s12916-017-0878-5

**Published:** 2017-05-31

**Authors:** Nicola Dalbeth, Lisa K. Stamp, Tony R. Merriman

**Affiliations:** 1 Department of Medicine, Faculty of Medical and Health Sciences, University of Auckland, 85 Park Rd, Grafton, Auckland 1023 New Zealand; 2 Department of Medicine, University of Otago, Christchurch, New Zealand; 3 Department of Biochemistry, University of Otago, Dunedin, New Zealand

**Keywords:** Gout, Urate, Genetics, Genome-wide association study, Personalised medicine

## Abstract

Over the last decade, there have been major advances in the understanding of the genetic basis of hyperuricaemia and gout as well as of the pharmacogenetics of urate-lowering therapy. Key findings include the reporting of 28 urate-associated loci, the discovery that *ABCG2* plays a central role on extra-renal uric acid excretion, the identification of genes associated with development of gout in the context of hyperuricaemia, recognition that *ABCG2* variants influence allopurinol response, and the impact of HLA-B*5801 testing in reducing the prevalence of allopurinol hypersensitivity in high-risk populations. These advances, together with the reducing cost of whole genome sequencing, mean that integrated personalised medicine approaches may soon be possible in clinical practice. Genetic data may inform assessment of disease prognosis in individuals with hyperuricaemia or established gout, personalised lifestyle advice, selection and dosing of urate-lowering therapy, and prevention of serious medication adverse effects. In this article, we summarise the discoveries from genome-wide association studies and discuss the potential for translation of these findings into clinical practice.

## Background

New discoveries regarding the genetic basis of hyperuricaemia and gout, gene-environment interactions, and the pharmacogenetics of urate-lowering therapy (ULT) have increased the possibilities for personalised medicine approaches to be used in clinical practice. In current clinical practice, genetic testing is relatively expensive and performed only where there is a strong clinical need in diagnosis or pharmacogenomics. However, the cost of generating the entire genome sequence for an individual is now less than US$ 1000 and falling. Therefore, future healthcare approaches may include the availability of a curated electronic whole genome sequence so that a genetic result can be available instantly. Herein, we provide an overview of the current knowledge generated by genome-wide association studies (GWAS), and discuss the potential for translation of these findings into integrated personalised medicine approaches for hyperuricaemia and gout.

## Genetics of hyperuricaemia and gout: recent discoveries

Serum urate levels and the risk of gout are influenced by a combination of inherited genetic variants and the environment. Heritability is defined as the percent variance in phenotype explained by inherited genetic variants, which can be estimated from studying phenotypic correlations between related individuals, typically twins. Such studies have estimated the heritability of urate to be between 45% and 73% [1–3]. In order to characterise the genetic basis of gout GWAS have been employed. A GWAS systematically assesses the genome for common (>1% prevalence) inherited genetic variants involved in disease etiology. These variants typically have a weak effect, with the majority exerting their effect by regulation of gene expression, transcript stability and transcript processing [4].

**Fig. 1:**
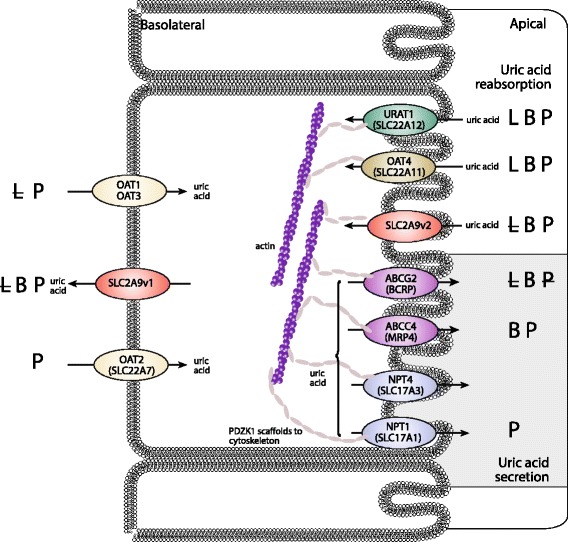
Influence of uricosuric agents lesinurad (L), benzbromarone (B) and probenecid (P) on the activity of renal uric acid transporters. Bold text, strong effect; normal text, weak to moderate effect; strikethrough, no effect; no text, no data were found. Based on information and data from [71–80]. *OAT* organic anion transporter; *URAT1* urate transporter 1; *NPT* sodium phosphate transporter. Adapted with permission from Dalbeth et al. [81]

In gout, the greatest insights have been derived from studies with serum urate as the outcome. The largest GWAS in Europeans involved 110,000 individuals and discovered 28 urate-associated loci [5], 10 of which had previously been reported in smaller GWAS [6–11]. These loci are dominated by genes encoding renal and gut uric acid transporters (*SLC2A9/GLUT9*, *ABCG2*, *SLC22A11/OAT4*, *SLC22A12/URAT1*, *SLC17A1/NPT1* and the auxiliary molecule *PDZK1*; Fig. 1). Statistically convincing sex effects are evident at the loci of strongest effect, with *SLC2A9* having a stronger effect in women and *ABCG2* in men [5]. The effects of the 28 single nucleotide polymorphisms (SNPs) were similar over multiple ancestral groups (European, African-American, Indian, Japanese) [5]. A GWAS of approximately 33,000 individuals in east Asians identified four loci (*SLC2A9*, *ABCG2*, *SLC22A12* and *MAF*) [12], all of which overlap with loci identified in Europeans. Considerably smaller GWAS have been performed in African-American sample sets [13, 14], with *SLC2A9* identified in both, with the larger study, that by Tin et al. [8], also detecting *SLC22A12* and a novel locus containing the *SLC2A12* and *SGK1* genes that encode GLUT12 and SGK1, respectively. A GWAS in the Micronesian population of Kosrae detected a genome-wide significant association with serum urate levels only at *SLC22A12* [15], and a very small GWAS in Mexican-Americans detected only *SLC2A9* [16]. Aside from the Micronesian study, these GWAS emphasise the relative importance of *SLC2A9* in the genetic control of urate across ancestral groups.

Aside from the uric acid transporter-containing loci the pathways influenced by the other loci remain largely speculative. The locus containing *GCKR*, encoding the glucokinase regulatory protein, may play a role in urate production via glycolysis (urate is produced as a result of ATP-depletion during hepatic glycolysis). Notably, none of the 18 loci that were newly identified by Köttgen et al. [5] encode known factors involved in uric acid transport. Several of these newly identified loci were within or near genes that encode transcription factors and growth factors, such as genes of the inhibin-activin growth factor network, *INHBB* and *ACVR2A*. One of the 18 newly identified loci includes *PRPSAP1*, which encodes a regulator of purine synthesis. Pathway analysis revealed that many of the 18 loci contained genes connected to glucose metabolism pathways. Emphasising the central role of hyperuricaemia in causing gout, the majority (24/28) of the urate-associated loci have been associated with gout in diverse populations [5, 17, 18]. Those with null associations are more likely a result of test sample sets of insufficient power than to a genuine lack of association, i.e. are false negatives.

*SLC2A9*, which encodes the GLUT9 protein, explains approximately 3% of variance in urate levels, a very large effect when compared to other complex disease loci. For example, the strongest effect in weight control in Europeans (the obesity associated protein FTO) explains only 0.3% of variance in phenotype [19]. The major *SLC2A9* genetic effect associates with isoform expression, whereby the urate-raising causal genetic variant associates with increased expression of an SLC2A9 isoform (SLC2A9-S) that has a 28-residue portion missing from the N-terminus [7, 8]. This isoform is expressed on the apical (urine) side of the renal tubular collecting duct, where it presumably increases reuptake of secreted uric acid, whereas the full-length version (SLC2A9-L) is expressed on the basolateral side, where it is the major basolateral exit route of uric acid into the blood [20]. There has been little progress in identifying functional candidate causal variants to date [21].

In contrast to the great majority of the urate-associated loci, the missense *rs2231142* (Q141K) variant in the *ABCG2* gene is highly likely to be causal with the 141 K variant reducing the ability of ABCG2 to secrete uric acid by approximately 50% [22]. Expression of most uric acid transporters is relatively high in the kidney or, for SLC22A12/URAT1, restricted to the kidney. However, expression of ABCG2 is also relatively high in the gut [23]. Matsuo et al. [24] created grades of ABCG2 dysfunction based on Q141K and Q126X (a second etiological variant in ABCG2) genotype combinations, with individuals positive for the dysfunctional variants 126X and 141 K having the highest serum urate levels and highest risk for gout. The presence of the 141 K (and 126X) alleles reduces excretion of uric acid through the gut and adds to the circulating urate, overloading the kidney excretion system, and resulting in increased urinary uric acid levels [25].

Rarer variants contributing to the etiology of gout do exist. However, owing to their scarcity they are statistically difficult to detect, unless they have a very strong effect on disease risk. Rare knockout variants in the *SLC2A9* and *SLC22A12* genes that block the reuptake of urinary filtered uric acid cause hypouricaemia and exercise-induced kidney failure [26–29]. Examples of uncommon (1–2%), but not rare, genetic variants associated with gout are a coding variant in the *ALDH16A1* gene in the Icelandic population (c.1580C > G; odds ratio, 3.7) [30], and the aforementioned knockout variant in the *ABCG2* gene in the Japanese population (Q126X; odds ratio, 4.3) [24]. The *ABCG2* 126X variant disables the uric acid export function of ABCG2; however, the molecular pathogenic basis of the *ALDH16A1* variant is unknown. In the coming years the possible contribution of rare penetrant functional variants in ABCG2 to gout will be evaluated [31]. Identification of further uncommon and rare population-specific variants will undoubtedly come from the study of whole genome sequences, although statistical confirmation will require very large sample sets comprising tens of thousands of people with gout.

GWAS in gout have only been performed in relatively small sample sets [5, 30, 32, 33], with the only novel loci reported in Chinese and Japanese GWAS. The Chinese GWAS used hyperuricaemic controls in follow-up testing to demonstrate that the newly discovered loci (*BCAS3*, *RFX3* and *KCNQ1*) are likely to be involved in pathways leading to presentation with gout in people with hyperuricaemia [32]. The potential role of candidate genes located at these novel loci in gout is not yet clear, although the *KCNQ1* association is notable. KCNQ1 is a potassium voltage-gated channel and an established type 2 diabetes susceptibility locus. The Japanese GWAS restricted controls to people with normouricaemia [33], making it more likely that the new loci reported (*MYL2-CUX2* and *CNIH2*) contribute to hyperuricaemia. A follow-up study from the Japanese GWAS identified *NIPAL1* (a magnesium transporter) and *FAM35A* (unknown function) [34]. Both are expressed in the distal tubules of the kidney, suggesting a role in uric acid handling [34].

Replicated candidate gene studies have identified some genes associated with gout. Most prominent are the *TLR4* gene and the *NLRP3* inflammasome component gene, both involved in the triggering of flares [35–37]. Interestingly, non-additive (epistatic) gene-gene interactions between the inflammasome *CARD8* C10X variant and a variant in *IL-1β* associated with IL-1β expression is consistent with an etiology where greater inflammasome activity from reduced CARD8 expression, combined with higher levels of pre-IL-1β expression, leads to increased production of mature IL-1β and an amplified immune response [37]. To our knowledge, the only other replicated candidate gene association in gout, but not serum urate levels, is with the apolipoprotein *A1-C3-A4* gene cluster [38, 39].

## Translation of genetic discoveries into clinical practice: towards personalised medicine for gout management?

In current clinical practice, there are a few situations for which genetic testing can assist with diagnosis and decisions about management. Monogenic syndromes such as partial hypoxanthine-guanine phosphoribosyltransferase deficiency (Kelley-Seegmiller syndrome), phosphoribosyl pyrophosphate synthetase overactivity, or autosomal dominant tubulointerstitial kidney disease caused by UMOD pathogenic variants are rare, and routine testing for these mutations is not required for the vast majority of individuals with gout. People with glucose-6-phosphate dehydrogenase deficiency are at risk of severe haemolysis and methemoglobinaemia when treated with pegloticase, and screening for this condition (typically by enzyme activity, rather than genotyping) is recommended before commencing pegloticase. In addition, human leukocyte antigen (HLA) variant HLA-B*5801 is an important risk factor for severe allopurinol hypersensitivity syndrome (AHS) [40], and therefore testing for this variant has been recommended in high risk populations (Han Chinese, other Asian populations) prior to commencing allopurinol [41].

These examples are isolated and specific, and current clinical practice does not routinely integrate genetic testing into gout management. A key question is how the new discoveries from GWAS of hyperuricaemia and gout can impact on clinical management of gout. Aside from identification of potential new therapeutic targets, genomic approaches may allow personalised assessment of prognosis, targeted lifestyle interventions, prediction of response to ULT, and prediction of adverse events to commonly used gout medications.

### Personalised assessment of prognosis

An important clinical question for individuals with hyperuricaemia is whether gout will develop, and for those presenting with gout, whether there is a risk of future flares and other severe consequences of disease such as tophi and/or joint damage [42]. A number of variables contribute to the risk of progressive disease, with serum urate levels being the most important variable identified to date [43]. However, serum urate levels alone do not reliably predict progression of disease. Risk stratification incorporating genetic testing may allow for more targeted decisions in an individual, for example, whether ULT should be initiated soon after (or even before) the first presentation of gout.

A consistent finding of GWAS has been the observation that, while *SLC2A9* variants are most strongly associated with hyperuricaemia, *ABCG2* variants are more strongly associated with gout [5]. In combination with evidence that *ABCG2* associates with gout using people with asymptomatic hyperuricemia as controls even after adjusting for baseline serum urate levels [44], it can be suggested that, in addition to its effects on serum urate, *ABCG2* variants may influence other checkpoints in the pathogenesis of gout, such as crystal formation and/or the inflammatory response to deposited crystals. The possibility that genetic testing has the potential to inform assessment of gout risk in people with hyperuricaemia is further supported by observations from the recent Chinese GWAS reporting three additional SNPs (*BCAS3*, *RFX3*, *PKCNQ1*) associated with gout when compared with asymptomatic hyperuricaemic controls [32].

Some studies have also implicated genetic variants in the development of severe manifestations of disease in those with established gout. A study from Aotearoa New Zealand reported that a non-synonymous *SLC2A9* Arg265His variant is associated with tophi in Māori with gout [45]. Two studies, one from Taiwan [42] and one from Aotearoa New Zealand [46, 47], have implicated *ABCG2* in the development of tophi in people with gout. In the Aotearoa New Zealand study [47], population-specific effects were observed, with several *ABCG2* SNPs (*rs2231142* (Q141K) and *rs10011796*) associated with tophi in Western Polynesian people with gout, independent of highest recorded urate and disease duration.

In order to clarify the importance of these findings to clinical practice, there is a need for large, well-characterised cohorts in different populations that follow individuals through the stages of disease, from hyperuricaemia to crystal deposition, to first presentation most often with flare, to advanced disease with tophus and chronic arthritis. Specifically, these studies will need to address the question of whether testing of genetic variants has additional benefit over standard clinical assessment, including urate levels, imaging assessment of urate deposition, and other known risk factors such as kidney function.

#### Targeted lifestyle interventions

Lifestyle changes are frequently advocated for prevention and management of gout [48]. Many different dietary changes are advocated, such as reducing intake of beer, sugar-sweetened drinks, and purine-rich foods such as meat, offal and seafood. Increased intake of cherries, omega-3 fatty acids, low fat milk and coffee are also advocated. At present, dietary recommendations are broad, generic and difficult to maintain in the long-term. Moreover, there is little evidence that such dietary changes actually influence serum urate levels in people with gout.

Some recent studies have reported gene-environment interactions in the regulation of serum urate levels or risk of gout. Examples include evidence for a non-additive interaction of sugar-sweetened drinks consumption with a urate-associated variant of *SLC2A9* in determining the risk of gout [49], and alcohol intake with *LRP2* in determining the risk of hyperuricaemia and gout [49–51]. A genetic risk score including *ABCG2*, *SLC2A9*, *SLC22A12*, *SLC22A11* and *SLC17A3* has also shown interaction with alcohol intake for gout risk [52].

An interaction between alcohol and the T allele of *LRP2 rs2544390* was initially described for serum urate levels in a Japanese cohort with the highest risk of hyperuricaemia in males with TT who consumed five or more drinks per week [51]. In a subsequent study from Aotearoa New Zealand [52], the T allele of *rs2544390* was also associated with increased risk of gout in a Polynesian cohort, but was associated with reduced risk of gout in a European cohort. There was a non-additive interaction between any alcohol intake and the risk of gout in the Polynesian cohort; any alcohol intake was associated with a 4.18-fold increased risk in the CC genotype group, compared with a 1.14-fold increased risk in the CT/TT genotype group. These effects were not observed in the European cohort [52].

The population-specific effects for the *LRP2*-alcohol interaction are instructive as they demonstrate that gene-environment findings in one population may not be translatable to other populations. A further, more fundamental issue is that lifestyle management (such as avoidance of sugar sweetened drinks or reduction of hazardous alcohol intake) may have health benefits beyond the risk of gout in individuals with genetic risk factors for gout. Any approach at personalised lifestyle advice based on genetic data will need to carefully address this issue to ensure that advice can be personalised to both gout and other comorbid conditions.

### Prediction of response to ULT

Identification of genetic variations that predict non-response to allopurinol and uricosurics brings the possibility of genetic testing to personalise selection of ULT. *ABCG2* is the only gene to date associated with non-response to allopurinol, the most widely used ULT agent. In 2015, a GWAS identified an association between the *ABCG2* 141 K allele and poor allopurinol response, defined by change in serum urate [53]. This association was replicated in a subsequent study with a stringent definition of poor response, namely serum urate levels ≥ 0.36 mmol/L despite allopurinol > 300 mg per day with adherence confirmed by plasma oxypurinol levels [54]. The 141 K allele frequency varies with ethnicity and ranges from 1% in African to 29% in South East Asian populations. Nevertheless, whether screening for 141 K and choosing alternative ULT in those with 141 K results in more rapid achievement of target urate remains to be determined. Further, the mechanism by which 141 K impairs allopurinol response remains unclear, although alterations in the transport of allopurinol and oxypurinol have been suggested [53].

Genetic variation in aldehyde oxidase (*AOX1*), encoding the enzyme responsible for the conversion of allopurinol to oxypurinol, and molybdenum cofactor sulfurase, also involved in the conversion, have been investigated. The minor allele of *AOX1 rs55754655* (N1135S) leads to a 2- to 4-fold greater AOX1 efficacy than wild-type AOX1, thereby conferring a fast metaboliser phenotype [55]. However, no association has been observed between *AOX1 rs55754655* (N1135S) and plasma oxypurinol levels or allopurinol dose required to achieve target serum urate [56]. Other genetic variants within *AOX1* and *XO* have also been reported to be associated with allopurinol dose and change in serum urate [57]. However, larger studies with a more robust definition of allopurinol response and correction for multiple testing are required.

Febuxostat is a ULT drug that also acts via xanthine oxidase inhibition. Febuxostat is metabolised in the liver by conjugation via uridine diphosphate glucuronosyltransferase enzymes and oxidation via cytochrome P450 (CYP) enzymes, including CYP1A2, CYP2C8 and CYP2C9. To date, there are no genetic studies examining febuxostat response. Febuxostat has been identified as an inhibitor of ABCG2 [58]. Whether this has implications for urate lowering and the effects of *ABCG2* SNPs on the urate lowering efficacy of febuxostat remains to be determined.

URAT1 (SLC22A12), which mediates the reabsorption of urate on the apical membrane of the proximal tubule in the kidney, is inhibited by the uricosurics probenecid, benzbromarone and lesinurad leading to normalisation of renal urate excretion [59, 60]. In addition, the renal urate transporters OAT1, OAT3, OAT4 (SLC22A11) and GLUT9 (SLC2A9) are variably inhibited by these agents (Fig. 1). There is some evidence that, in people with renal hypouricaemia, a loss of function mutation in *URAT1* (W258X; *SLC22A12 774G > A*) is associated with impaired response to probenecid and benzbromarone [61]. Thus, genetic variants associated with hyperuricaemia and gout may also have pharmacogenetic relevance.

### Prediction of adverse effects to commonly used gout medications

The identification of HLA-B*5801 as a major risk factor for AHS has been a major advance in safe prescribing of this medication. This test is now established in clinical practice in high-risk populations, and implementation of this screening has led to a reduction in the prevalence of this life-threatening complication [62].

In European people, HLA-B*5801 is a strong risk factor for AHS but is not sufficient or necessary to explain the disease, in contrast to Han Chinese and other Asian populations [63]. A GWAS of European AHS cases identified a six SNP haplotype (CACGAC) that was significantly associated with AHS (odds ratio, 7.77) [64]. The SNPs in this haplotype are located within the HLA locus on chromosome 6, but are only in partial linkage disequilibrium with HLA-B*5801. In a large Aotearoa New Zealand gout cohort, the CACGAC haplotype occurred at a higher frequency in European patients who experienced mild (non-AHS) allopurinol-related adverse events (13.3% vs. 1.7%; odds ratio, 8.9), but was not associated with allopurinol-related adverse events in Polynesians [65]. These data highlight the potential utility of genetic testing to also predict milder adverse effects in patients on ULT.

Another example of genetic testing with the potential to assess the risk of adverse effects to ULT is testing the cytochrome CYP2C9 poor metaboliser alleles CYP2C9*2 and CYP2C9*3. CYP2C9*3 homozygotes have a markedly longer benzbromarone elimination half-life than other *CYP2C9* genotypes, which may increase the risk of benzbromarone-induced hepatotoxicity [66]. The frequency of CYP2C9 poor metaboliser alleles is substantially higher in Europeans compared to Polynesians [67]. Given the role of CYP2C9 in the metabolism of febuxostat, it is possible that those with the poor metaboliser allele are at risk of adverse effects with febuxostat; this is worthy of further investigation.

Genetic testing might also guide the choice of anti-inflammatory medications for flare management. CYP2C9 also metabolises many non-steroidal anti-inflammatory drugs (NSAIDs), including celecoxib, diclofenac, ibuprofen, naproxen and piroxicam. In a study of acute NSAID users, endoscopically documented NSAID-related gastroduodenal bleeding lesions were more common in those with poor metaboliser alleles [68].

### Future directions and conclusions

Rapidly progressive technology and disease-specific genetic discoveries have the potential to make personalised medicine a reality in many aspects of gout management, including risk assessment of disease progression, personalised lifestyle advice, selection and dosing of ULT, and prevention of serious medication adverse effects. Although major progress has been made through GWAS, there is a further need for large, well-characterised datasets that include different disease states, detailed pharmacology (including dose information, treatment response, adverse drug reactions) and lifestyle information. A further challenge is population-specific effects, meaning that discoveries in one population may not be translatable to other populations. In order to avoid increasing the disparities that are already evident in gout management [69, 70], study of different populations will be essential, particularly of those with high prevalence of severe disease.

## Abbreviations

AHS: allopurinol hypersensitivity syndrome
AOX: aldehyde oxidase
CYP: cytochrome P450
GWAS: genome-wide association studies
HLA: human leukocyte antigen
NSAIDs: non-steroidal anti-inflammatory drugs
SNP: single nucleotide polymorphism
ULT: urate-lowering therapy
